# Machine Learning Approaches for Predicting Intraoperative Blood Transfusion in Partial Hip Arthroplasty

**DOI:** 10.3390/jcm14217657

**Published:** 2025-10-28

**Authors:** Mürsel Kahveci

**Affiliations:** Anesthesiology and Reanimation, Amasya Training and Reserch Hospital, Amasya University, 05100 Amasya, Turkey; drmurselkahveci@yahoo.com; Tel.: +90-506-2791639; Fax: +90-358-2600070

**Keywords:** partial hip arthroplasty, machine learning, prediction model, transfusion

## Abstract

**Objective:** Partial hip arthroplasty (PHA) procedures are often associated with significant blood loss, particularly in elderly patients with comorbidities. Predicting the need for intraoperative transfusion in advance is crucial for patient safety and surgical planning. Machine learning (ML) algorithms offer data-driven solutions to support clinical decision-making in such scenarios. **Methods:** This retrospective, single-center cohort study evaluated data from 202 patients who underwent PHA between December 2023 and July 2025. Demographic data, as well as preoperative and intraoperative variables, were collected. Six ML algorithms—Logistic Regression, Decision Tree, Support Vector Machines (SVM), Artificial Neural Network (ANN), Random Forest, and Gradient Boosting—were trained and tested to predict intraoperative blood transfusion. Model performance was assessed using accuracy, F1-score, and area under the ROC curve (AUC). SHAP (SHapley Additive exPlanations) analysis was used to evaluate model interpretability. **Results:** Among the 202 patients, 85 (42.1%) received intraoperative blood transfusions. Significant predictors included low preoperative hemoglobin, high ASA score, prolonged operative time, increased intraoperative blood loss, and elevated INR (all *p* < 0.05). The Random Forest and Decision Tree models achieved the highest accuracy (95.1%) and F1-score (0.960), while the SVM model yielded the highest AUC (0.992). SHAP analysis identified hemoglobin, age, ASA score, INR, and operative time as the most influential features in model decision-making. **Conclusions:** Machine learning models—particularly Random Forest, Decision Tree, and SVM—demonstrated high performance in predicting intraoperative transfusion needs during PHA. The incorporation of explainable AI techniques such as SHAP enhanced the clinical interpretability of model outputs, supporting personalized patient management. These findings provide a strong foundation for integrating such models into clinical decision support systems, though external validation through multicenter and prospective studies is warranted.

## 1. Introduction

Partial hip arthroplasty (PHA) is an effective orthopedic surgical method commonly applied to elderly individuals following femoral neck fractures, advanced osteoarthritis, avascular necrosis, or traumatic injuries. Its primary goal is to restore functional independence and improve quality of life [1]. However, these operations are frequently associated with significant blood loss due to factors such as advanced age, comorbid conditions, and anatomical changes. Both intraoperative and postoperative bleeding can disrupt hemodynamic stability, prolong recovery time, increase morbidity, and often necessitate blood transfusion [2,3].

Although transfusion is sometimes essential for maintaining vital functions during surgical procedures, it carries risks such as infection, acute kidney injury, immunological reactions, and prolonged hospital stay [4,5]. Therefore, preoperative prediction of transfusion requirements in PHA patients—especially elderly individuals with systemic diseases is critically important for both patient safety and optimal resource utilization within the healthcare system [6]. Current conventional blood preparation protocols are largely based on standard guidelines and the clinical experience of the surgeon. However, this approach may fail to adequately reflect individual patient differences and the specific dynamics of the surgery [7]. Thus, there is a growing need for objective and data-driven methods that can more accurately estimate transfusion requirements.

Traditional statistical methods often fall short in predicting transfusion needs due to their limited capacity to capture the heterogeneity of patient populations and complex multivariate relationships [8]. Unlike traditional statistical approaches, ML models have non-constant variance, complex feature interactions, and nonlinear dependencies due to the tree-bas sRando, Gradient, and CatBo, which have demonstrated powerful ML for classical statistical methods, offering the ability to extract meaningful patterns from large and multidimensional datasets and to model nonlinear and high-order relationships that conventional regression models often fail to capture [9,10]. Recent studies have successfully implemented various ML algorithms, including Support Vector Machines (SVM), Decision Trees (DT), Artificial Neural Networks (ANN), XGBoost, LightGBM, and CatBoost, to predict intraoperative and postoperative transfusion requirements, demonstrating their superior predictive accuracy compared to traditional logistic regression models [2,8,11,12,13,14,15]. These models not only provide improved discrimination and calibration performance but also contribute to the enhancement of clinical decision support systems by integrating multiple clinical, biochemical, and perioperative variables into a unified predictive framework [16]. Therefore, ML-driven predictive modeling has the potential to support individualized patient management, optimize transfusion strategies, and ultimately improve surgical outcomes. Moreover, the application of SHAP (Shapley Additive Explanations) analysis, particularly in XGBoost models, has enhanced interpretability by visually identifying the most influential factors in transfusion decisions [11,17]

The literature indicates that major predictors of transfusion in orthopedic surgery include preoperative hemoglobin level, age, body mass index (BMI), platelet count, ASA score, hypertension, diabetes, operative duration, international normalized ratio (INR), intraoperative blood loss, and use of tranexamic acid (TXA) [6,18,19]. Studies conducted in total hip arthroplasty (THA) and other major orthopedic procedures have confirmed the significant contribution of these factors to transfusion risk. Additionally, models developed by Han Zang et al. have shown that incorporating specific risk factors from PHA cases improves model performance, underscoring the importance of tailored prediction models for this patient population [18].

Most existing machine learning models in the literature have focused on total hip arthroplasty and other large orthopedic surgeries, with limited emphasis on the specific characteristics of partial hip arthroplasty cases. This highlights a significant gap in the literature and the potential value of developing dedicated transfusion prediction models for PHA. The aim of this study is to develop a machine learning model capable of predicting intraoperative blood transfusion requirements in patients undergoing PHA. The model will utilize preoperative and intraoperative clinical and laboratory data, and compare the performance of SVM, DT, and ANN algorithms in terms of sensitivity and specificity. Ultimately, early identification of high-risk patients will enable the implementation of targeted preventive strategies, minimizing transfusion needs and associated complications. Furthermore, accurate predictions can help reduce unnecessary blood reservations and prevent waste of healthcare resources.

## 2. Materials and Methods

### 2.1. Study Design

This study was designed as a retrospective, single-center, observational cohort study. Preoperative, intraoperative clinical, and laboratory data of patients who underwent PHA surgery at Amasya University Hospital were examined. The study was conducted in accordance with the principles of the Declaration of Helsinki and received approval number 2025/168 from the Amasya University Clinical Research Ethics Committee. The primary objective of the study was to develop a machine learning model that can predict the need for intraoperative blood transfusion during PHA.

### 2.2. Patient Characteristics

This study includes data from patients who underwent PHA at the Amasya University Faculty of Medicine between December 2023 and July 2025. Patients with pathological fractures; multiple fractures or polytrauma; those who did not receive surgical treatment or had missing clinical data were excluded. Additionally, individuals with trauma or surgery that could alter bone structure were also excluded.

Inclusion criteria consisted of patients with unilateral fractures resulting from low-energy trauma, with no contraindications to surgery, who underwent standard preoperative blood work (including routine labs, immune function, coagulation, and liver/kidney function tests), and who received partial hip arthroplasty (hemiarthroplasty).

Out of 600 patient records reviewed retrospectively, 202 patients meeting the inclusion criteria were included in the study. The mean age of the study population was 74.8 ± 6.2 years. Of these, 134 (66.3%) were female and 68 (33.7%) were male. Intraoperative blood transfusion was administered to 85 patients (42.1%), while 117 patients (57.9%) did not require transfusion.

Table 1 presents the demographic characteristics, comorbidities, and preoperative evaluation findings of the study population. In the transfused group (n = 85), 25 were male (29.4%) and 60 were female (70.6%), while in the non-transfused group (n = 117), 43 were male (36.8%) and 74 were female (63.2%). Variables such as age, BMI, hypertension, diabetes mellitus, coronary artery disease, ASA score, and aspirin use were also compared to identify potential clinical indicators associated with transfusion need.

**Figure 1:**
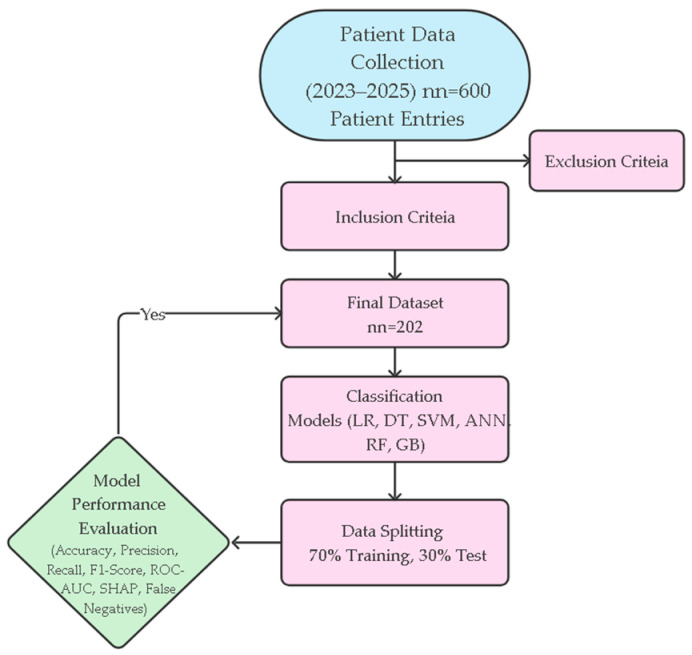
Study Flowchart.

All descriptive statistics and intergroup comparisons are shown in Table 1. The methodological workflow of the study from data collection to model development and performance evaluation is systematically summarized in Figure 1. This flowchart holistically reflects all stages of the research, including patient selection, data preprocessing, modeling, and performance evaluations.

Perioperative blood transfusion indications in our institution are standardized. Transfusion is recommended in the presence of significant clinical signs of anemia such as tachycardia, hypotension, or shock, or when the hemoglobin level falls below 80 g/L regardless of symptoms. The final decision on transfusion is made jointly by the surgeon and anesthesiologist, considering both laboratory and clinical findings.

Patients’ preoperative laboratory findings and intraoperative characteristics were compared according to transfusion status and are detailed in Table 2. Preoperative parameters included hemoglobin (Hgb), platelet count, white blood cell count, and international normalized ratio (INR). Intraoperative variables included surgical duration, estimated blood loss, pre-induction systolic and diastolic blood pressure, and calculated mean arterial pressure. Surgical planning parameters included prosthesis type (cemented vs. uncemented) and type of anesthesia (spinal vs. general). The primary outcome variable was defined as the number of blood units transfused intraoperatively. All surgical data were obtained from anesthesia records and surgical notes. An independent samples *t*-test was used for continuous variables, and a chi-square or Fisher’s exact test was used for categorical variables. Statistical significance was set at *p* < 0.05.

### 2.3. Statistical Analysis

Descriptive and analytical statistics were performed using IBM SPSS Statistics 28.0 (IBM Corp., Armonk, NY, USA). A *p*-value < 0.05 was considered statistically significant. Continuous variables were reported as mean ± standard deviation, and categorical variables were expressed as frequencies and percentages. Between-group comparisons were conducted using an independent samples *t*-test for continuous variables and chi-square test (or Fisher’s exact test where appropriate) for categorical variables. These statistical tests were used exclusively for the descriptive comparisons presented in Table 1 and Table 2, and are independent of the machine learning modeling process. Statistical results were presented in Table 1 (demographics and preoperative features) and Table 2 (intraoperative features).

### 2.4. Classification

Classification modeling was conducted using Python (version 3.10) with open-source libraries such as scikit-learn, NumPy, pandas 2.0.1, matplotlib, and SHAP. The main goal was to develop a reliable and explainable machine learning model to predict intraoperative blood transfusion (target variable: 0 = no transfusion, 1 = transfusion) based on preoperative and intraoperative parameters.

During preprocessing, categorical variables (e.g., sex) were transformed using One-Hot Encoding. Continuous variables were normalized using z-score standardization to eliminate scale sensitivity. The dataset was randomly split into 70% training (n = 141) and 30% test (n = 61) sets. The “stratify” parameter was used to preserve class distribution, and 5-fold stratified cross-validation was applied for model evaluation.

Six classification algorithms were compared: Logistic Regression, Decision Tree, Support Vector Machine (SVM), Multilayer Perceptron (MLP), Random Forest, and Gradient Boosting. For SVM, the Radial Basis Function (RBF) kernel was used; the decision tree was optimized using both “Gini” and “Entropy” criteria. The neural network model had a single hidden layer (100–50 neurons) with ReLU activation and Adam optimizer. The Random Forest and Gradient Boosting models were configured with 200 estimators.

Model performance was assessed using accuracy, precision, recall, specificity, F1-score, area under the ROC curve (AUC), and cross-validation mean ± standard deviation. SHAP (SHapley Additive exPlanations) analysis was applied to enhance the clinical interpretability of model outputs and visualize the impact of features on predictions. ROC curves and cross-validation standard deviations were also used to compare performance across algorithms.

## 3. Results

In this study, machine learning models were developed to predict the need for intraoperative blood transfusion using preoperative and intraoperative data from 202 patients who underwent partial hip arthroplasty (PHA), and the classification performance of these models was compared.

Demographic and clinical differences between the transfused group (n = 85) and the non-transfused group (n = 117) are presented in Table 1 and Table 2. The mean age was significantly higher in the transfused group (76.0 ± 6.3 vs. 73.9 ± 6.0; *p* = 0.015), and comorbidities such as hypertension, diabetes mellitus, and coronary artery disease were significantly more prevalent (*p* = 0.007, *p* = 0.019, and *p* = 0.003, respectively). Significant differences were also observed in preoperative parameters including ASA score, aspirin use, hemoglobin level, platelet count, and INR values. In the intraoperative period, surgical duration, estimated blood loss, and arterial blood pressure values were notably higher in the transfused group (all *p* < 0.05). Additionally, cemented prosthesis and general anesthesia were more commonly used in this group.

Six different machine learning models (Logistic Regression, Decision Tree, SVM, Neural Network, Random Forest, Gradient Boosting) were trained using the collected data and evaluated on the test set. Their performance metrics are summarized in Table 3. The models with the highest accuracy and F1-score were the Decision Tree and Random Forest (accuracy = 0.951, F1 = 0.960). In terms of area under the ROC curve (AUC), the SVM model achieved the best performance (AUC = 0.992). According to 10-fold cross-validation, the Random Forest model demonstrated the highest average accuracy with the lowest standard deviation (CV = 0.984 ± 0.009), indicating strong model stability.

Specifically, the ability to predict transfusion need using preoperative hemoglobin, ASA score, INR, and intraoperative parameters allows clinicians to optimize blood reservation strategies, thereby reducing unnecessary crossmatching and preserving blood bank resources. Furthermore, identifying modifiable risk factors such as low hemoglobin levels, elevated INR, and the discontinuation of ASA use within 24 h can guide preoperative optimization protocols. This may reduce, or even eliminate, the need for transfusion in selected patients, ultimately contributing to enhanced patient safety, reduced complications, and more efficient resource utilization. These findings underline the potential clinical relevance of our model, despite the limitations in study design and sample size planning.

**Figure 2:**
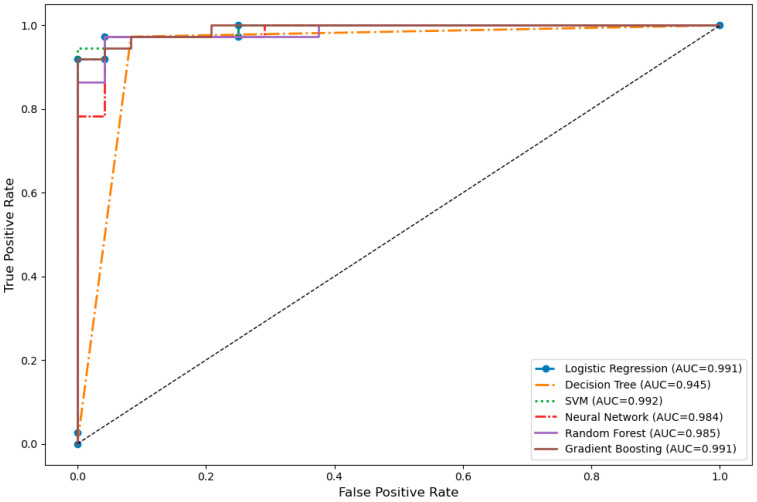
Comparison of ROC curves for different machine learning models.

The visual analysis of the models’ discriminative power is presented in Figure 2 through ROC curves. The SVM, Logistic Regression, and Gradient Boosting models demonstrated superior performance in terms of AUC values, while the Decision Tree algorithm exhibited relatively limited discriminative power with a lower AUC of 0.945. Overall, the ROC curves revealed that all models possessed high sensitivity and specificity.

**Figure 3:**
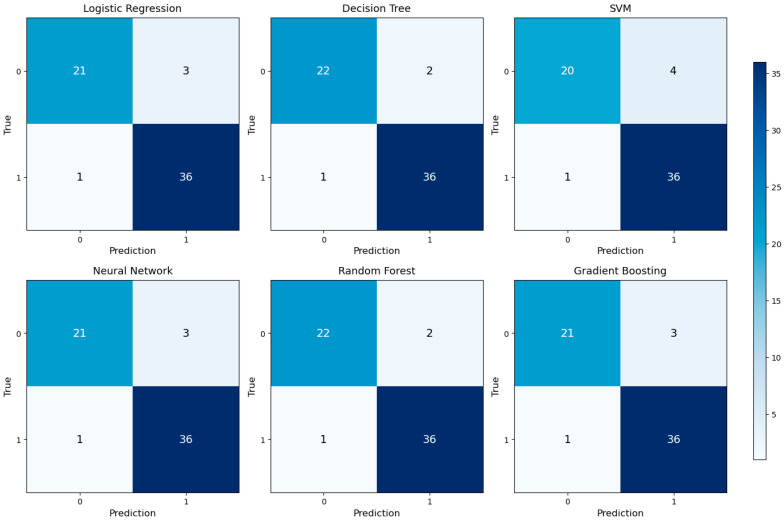
Confusion matrix results for six different machine learning models.

Classification accuracies were further detailed using confusion matrices, as shown in Figure 3. Each model accurately classified true positive and true negative cases, with minimal false negative rates. This indicates that the models are reliable, especially in identifying patients who truly require transfusion in clinical practice.

**Figure 4:**
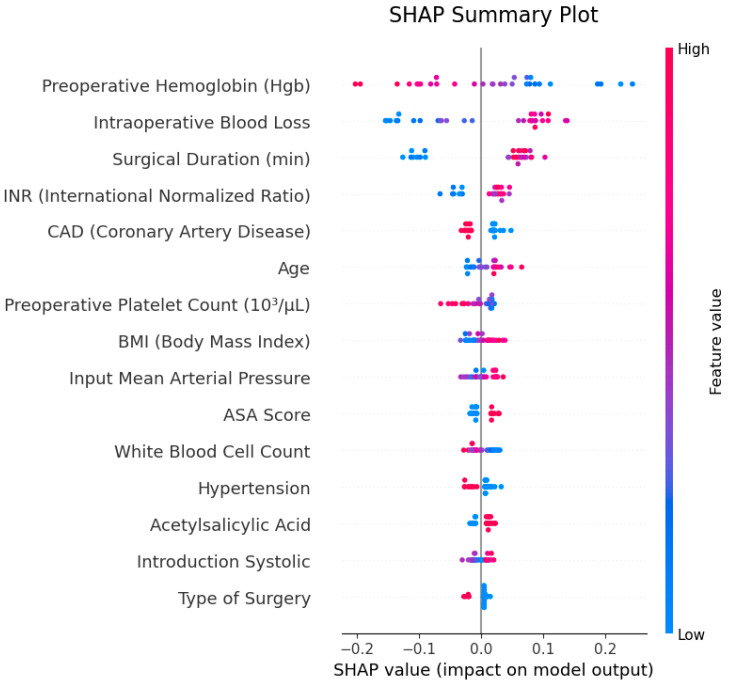
SHAP interaction analysis showing the bidirectional interaction between “gender” and “age” features.

To evaluate the explainability of model outputs, SHAP (SHapley Additive exPlanations) analysis was applied, and the results are illustrated in Figure 4. The SHAP summary plot identified “preoperative hemoglobin (preop Hgb)”, “intraoperative blood loss” and “surgical duration (min)” as the most influential features in the model’s decision-making process. The SHAP values of these features contributed both positively and negatively to predictions, with low hemoglobin levels and high blood loss standing out as primary factors increasing transfusion probability.

In the SHAP feature importance plot, the average contribution of features to the model was visualized, confirming that the same three variables were most prominent. These findings demonstrate that the model bases its decisions on clinically meaningful and interpretable parameters.

**Figure 5:**
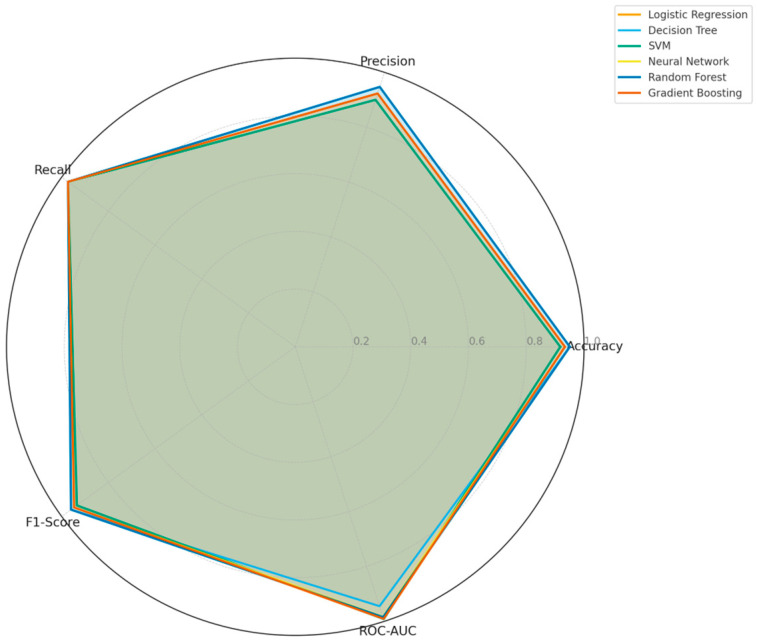
Radar chart comparing six machine learning algorithms based on Accuracy, Precision, Recall, F1-Score, and ROC-AUC metrics.

Finally, a radar chart presented in Figure 5 was used to compare the multi-dimensional classification performances of all models. The chart provides a holistic view of Accuracy, Precision, Recall, F1-Score, and ROC-AUC metrics. Overall, SVM, Logistic Regression, and Gradient Boosting performed best in ROC-AUC and recall, whereas Random Forest and Decision Tree models showed stronger results in terms of F1-score and accuracy. This analysis highlights the importance of multi-criteria optimization rather than relying on a single metric when selecting the optimal model.

## 4. Discussion

In this study, we evaluated six machine learning models to predict intraoperative blood transfusion requirement in patients undergoing partial hip arthroplasty (PHA). Our results indicate that Random Forest (RF), Decision Tree (DT), and Support Vector Machine (SVM) models performed particularly well. RF and DT achieved strong classification metrics with accuracy of 95.1% and F1-score of 0.960, while the SVM model achieved the highest discriminative capacity in terms of ROC-AUC (0.992). These findings align with prior studies that underscore the superior classification performance of Random Forest in clinical prediction tasks [2,20].

Notably, the RF model exhibited remarkable stability, with a low standard deviation (±0.009) in cross-validation, supporting its generalizability. Such stability may be attributed to the ability of tree-based models to capture nonlinear relationships and manage noisy, high-dimensional clinical data [2,20]. For instance, Zhu et al. developed a dynamic model predicting transfusion risk after total hip surgery and also found that surgical bleeding, preoperative hemoglobin, and surgery type were prominent risk factors [21]. Similarly, in the study by Zang et al. on perioperative transfusion prediction in hip surgery, 14 models were evaluated on 2431 cases; low hemoglobin, high ASA score, low fibrinogen, and prolonged operative time emerged as key predictors [18]. In our work, preoperative hemoglobin, ASA score, and surgical duration also ranked among the most influential factors. Furthermore, the robust AUC performance of the SVM model demonstrates that even with a limited sample size, strong discriminative ability can be achieved. Zhu et al. (2024) similarly highlighted Random Forest’s performance in femoral neck fracture patients [21]; while Chen et al. (2023) reported superior results for Gradient Boosting in total hip arthroplasty datasets [2]. Guo et al. (2024) also found that both Random Forest and Logistic Regression provided strong transfusion prediction in hip fracture populations [22].

Key predictors in our models included preoperative hemoglobin, ASA score, age, INR, and surgical duration. The role of low hemoglobin is mechanistically plausible: it limits intraoperative oxygen delivery capacity, increasing the likelihood of transfusion [23,24]. This observation is echoed in works by Spahn (2020) and Zhou et al. (2024) [3,20]. The ASA score reflects the overall health and surgical risk of the patient, contributing significantly to risk stratification [25].

We also found that patients with longer surgical durations and greater intraoperative blood loss had significantly higher likelihoods of receiving transfusion. This underscores the impact of intraoperative dynamics on postoperative transfusion requirements. Surgical time often correlates with procedural complexity, surgeon experience, and anatomical variation. Prolonged surgical duration increases opportunities for bleeding—both visible and hidden—and thus predisposes to transfusion needs [21]. Surace et al. (2023) reported a marked increase in postoperative transfusion incidence when operative time exceeded 75–80 min [26]. Similarly, Cai et al.’s retrospective review of 707 total hip arthroplasty cases found an average hidden blood loss of 700.39 ± 368.59 mL [27]. This evidence suggests that surgical procedures contribute not only to visible bleeding but also to significant hidden (non-apparent) losses. In total hip arthroplasty, hidden losses may account for up to ~50% of total blood loss [8]. Because hip arthroplasty often cannot use a tourniquet, soft tissue and bony disruption may lead to such hidden losses. Consequently, we employed the Gross formula in our study to estimate total blood loss—including both intraoperative and potential hidden components—as a means to improve the accuracy of transfusion requirement prediction [21].

To enhance interpretability, we applied SHAP (SHapley Additive exPlanations) analysis. This approach helps to demystify the “black box” nature of AI models by elucidating feature-level contributions. Our SHAP results showed that preoperative hemoglobin, intraoperative blood loss, and surgical duration were the top drivers of model decisions. In particular, lower hemoglobin and higher blood loss increased the predicted probability of transfusion [20,21]. These findings align with recent work in critical care and surgical settings. For example, Sheikhalishahi et al. (2024) developed a machine learning model for post-surgical ICU patients and identified hemoglobin, age, surgical duration, and coagulation parameters as important predictors [28]. Similarly, Duranteau et al. (2024) in their review emphasize that integrating SHAP or similar explainable AI techniques enhances the trustworthiness and clinical applicability of prediction models [25].

Overall, our SHAP analysis outcomes reinforce that our machine learning models rely not only on statistical associations but on variables with clear clinical significance. The presence of recognizable predictive factors such as hemoglobin, surgical bleeding, and operative time bolsters the interpretability and trustworthiness of the model outputs. This interpretability supports the development of individualized transfusion strategies, more efficient planning of blood product allocation, and enhanced patient safety in the perioperative setting.

However, it is important to note that SHAP values do not quantify the proportion of total variance in the outcome explained by each feature, as traditional statistical models do. While they enhance local interpretability, they do not provide a global variance decomposition. Therefore, although SHAP supports clinical transparency and decision-making, it does not replace formal statistical attribution models in terms of explanatory power.

## 5. Limitations

This study has several limitations. First, it was conducted retrospectively in a single-center setting, which may introduce selection bias and limit the generalizability of the findings. Although our machine learning models performed well internally, their validity in external populations remains untested. Second, the range of predictor variables was restricted to routinely available clinical and laboratory data. More detailed intraoperative monitoring parameters and perioperative interventions—such as TXA administration, preoperative anemia management, and postoperative bleeding indicators—were not consistently documented and therefore excluded. Third, transfusion decisions may have been influenced by individual clinician judgment, adding subjectivity to the outcome definition. Future studies should incorporate these missing variables through prospective, multicenter designs with standardized data collection to enhance model robustness, external validity, and clinical applicability.

## 6. Conclusions

This study demonstrates that machine learning models can predict intraoperative transfusion needs in partial hip arthroplasty patients with high accuracy and interpretability. In particular, Random Forest, Decision Tree, and SVM models achieved robust performance across accuracy, ROC-AUC, and F1-score metrics, highlighting their potential for integration into clinical decision support systems. The top predictive features—preoperative hemoglobin, intraoperative blood loss, and surgical duration—are clinically interpretable and consistent with existing literature. The use of SHAP as an explainable AI technique further enhances the clinical trustworthiness of model predictions and supports personalized patient care. These findings suggest meaningful benefits in preoperative identification of high-risk patients, minimizing unnecessary transfusions, reducing complications, and optimizing resource utilization. Future directions include external validation with multicenter, prospective, real-time data and seamless integration of AI support into clinical pathways.

## Figures and Tables

**Table 1:** Comparison of Demographic and Clinical Characteristics Based on Transfusion Status.

Variable	All Patients (n = 202)	Non-Transfusion Group (n = 117)	Transfusion Group (n = 85)	*p*-Value
Demographic Characteristics				
Age (years)	74.8 ± 6.2	73.9 ± 6.0	76.0 ± 6.3	0.015 *
Gender, n (%)				0.266
Female	134 (66.3)	74 (63.2)	60 (70.6)	
Male	68 (33.7)	43 (36.8)	25 (29.4)	
Body Mass Index (kg/m^2^)	31.4 ± 2.7	31.2 ± 2.6	31.7 ± 2.8	0.185
Comorbidities				
Hypertension, n (%)	128 (63.4)	65 (55.6)	63 (74.1)	0.007 *
Diabetes Mellitus (DM), n (%)	56 (27.7)	25 (21.4)	31 (36.5)	0.019 *
Coronary Artery Disease (CAD), n (%)	94 (46.5)	44 (37.6)	50 (58.8)	0.003 *
Preoperative Assessment				
ASA Score, n (%)				<0.001 *
ASA 2	116 (57.4)	87 (74.4)	29 (34.1)	
ASA 3	86 (42.6)	30 (25.6)	56 (65.9)	
ASA Discontinued ≥ 24 h Preop, n (%)	105 (52.0)	53 (45.3)	52 (61.2)	0.026 *

* Statistically significant at *p* < 0.05.

**Table 2:** Comparison of Laboratory and Intraoperative Features Based on Transfusion Status.

Variable	All Patients (n = 202)	Non-Transfusion Group (n = 117)	Transfusion Group (n = 85)	*p*-Value
Preoperative Laboratory				
Preop Hemoglobin (g/dL)	11.4 ± 0.9	11.9 ± 0.7	10.7 ± 0.7	<0.001 *
Preop Platelets (10^3^/µL)	228.2 ± 37.5	232.8 ± 36.1	221.9 ± 38.2	0.036 *
Preop Leukocytes (10^3^/µL)	7.5 ± 1.6	7.4 ± 1.6	7.6 ± 1.6	0.401
Preop INR	1.08 ± 0.11	1.05 ± 0.09	1.12 ± 0.11	<0.001 *
Intraoperative Parameters				
Surgery Duration (minutes)	76.5 ± 15.2	71.8 ± 12.4	82.9 ± 15.9	<0.001 *
Estimated Blood Loss (mL)	553.2 ± 116.5	493.6 ± 81.2	635.1 ± 108.3	<0.001 *
Initial Systolic BP (mmHg)	129.8 ± 10.5	128.1 ± 10.1	132.1 ± 10.5	0.005 *
Initial Diastolic BP (mmHg)	85.2 ± 8.9	84.1 ± 8.7	86.7 ± 9.0	0.034 *
Initial Mean Arterial Pressure (mmHg)	108.9 ± 10.2	107.1 ± 9.8	111.4 ± 10.2	0.002 *
Surgical Characteristics				
Type of Prosthesis, n (%)				0.002 *
Cementless	35 (17.3)	28 (23.9)	7 (8.2)	
Cemented	167 (82.7)	89 (76.1)	78 (91.8)	
Type of Anesthesia, n (%)				0.004 *
Spinal	170 (84.2)	106 (90.6)	64 (75.3)	
General	32 (15.8)	11 (9.4)	21 (24.7)	

* Statistically significant at *p* < 0.05.

**Table 3:** Comparison of Performance Metrics for Machine Learning Models.

Model	Accuracy	Precision	Recall	F1-Score	ROC-AUC	CV Mean ± STD
Logistic Regression	0.934	0.923	0.973	0.947	0.991	0.981 ± 0.011
Decision Tree	0.951	0.947	0.973	0.960	0.945	0.896 ± 0.018
SVM	0.918	0.900	0.973	0.935	0.992	0.977 ± 0.022
Neural Network	0.934	0.923	0.973	0.947	0.984	0.981 ± 0.014
Random Forest	0.951	0.947	0.973	0.960	0.985	0.984 ± 0.009
Gradient Boosting	0.934	0.923	0.973	0.947	0.991	0.977 ± 0.013

## Data Availability

The original contributions presented in this study are included in the article. Further inquiries can be directed to the corresponding author.
